# Association between the Prevalence of Metabolic Syndrome and the Level of Coffee Consumption among Korean Women

**DOI:** 10.1371/journal.pone.0167007

**Published:** 2016-12-15

**Authors:** Keyhoon Kim, Kyuwoong Kim, Sang Min Park

**Affiliations:** 1 Department of Medicine, Seoul National University College of Medicine, Yunkeon-dong, Jongro-gu, Seoul, South Korea; 2 Department of Biomedical Sciences, Seoul National University Graduate School, Biomedical Science Building, Daehakro, Jongro-gu, Seoul, South Korea; 3 Department of Family Medicine, Seoul National University Hospital, Seoul National University College of Medicine, Daehak-ro, Jongno-gu, Seoul, South Korea; University of Hawai'i at Manoa College of Tropical Agriculture and Human Resources, UNITED STATES

## Abstract

**Background:**

As coffee consumption is increasing remarkably over the past decade, the health effects concerning the coffee drinking has gained a wide attention across the nation. However, there is not a true consensus regarding the effects of coffee on metabolic disease. Therefore, this study aims to examine the association between coffee intake and the risk of metabolic syndrome in Korean women

**Methods:**

We used publicly accessible datasets collected through Korean National Health and Nutrition Examination Survey (KNHANES). Among 20,435 individuals from five consecutive years’ worth of data from 2007 to 2011, only 15,691 subjects qualified for statistical analysis upon applying the exclusion criteria. We carried out the statistical analysis utilizing SPSS Statistics version 13.0 (IBM Corp., Armonk, NY.) and STATA statistical software release 13.0 (STATA Corp., College Station, TX).

**Results:**

We found that the frequency of coffee intake inversely correlates with the prevalence of metabolic syndrome in women. Upon adjusting for life-style factors, socioeconomic status, and nutritional profile, the subjects from the highest coffee consumption quartile exhibited 40% lower odds of suffering from metabolic syndrome compared to those in the control (OR = 0.75; 95% CI = 0.67–0.84; P for trend < 0.001). Also, we observed that age- and BMI-adjusted HOMA-IR decreased as the coffee consumption increased (P for trend < 0.001).

**Conclusion:**

The findings of our study suggest that coffee consumption might be associated with reduction of metabolic syndrome in Korean women. To elucidate this cross-sectional association between coffee consumption and metabolic syndrome in women, cohort studies are warranted to confirm this relationship.

## Introduction

Metabolic syndrome is a cluster of complications that could increase the risk of atherosclerotic cardiovascular disease (CVD) through a shared pathophysiology. In the Republic of Korea, the metabolic syndrome has become a major public health issue, as the prevalence of metabolic syndrome in Korea is increasing at a rapid rate. [1] Many physicians and scientists across the world are trying to discover the underlying mechanism of metabolic syndrome because these CVD risks tend to cluster in a patient. [2] Insulin resistance is one of the hall marks of metabolic syndrome. It has been suggested not only as a principal initiator of atherogenic inflammation, but also as a perpetuator in the final common pathway of metabolic alteration. [3] Some previous studies have suggested the potential association between coffee intake and the risk of metabolic syndrome. [4–6]

Coffee is one of the most widely consumed beverages around the world. Especially in Korea, coffee consumption has increased remarkably over the past decade. As a result, health effects concerning coffee drinking has gained attention across the world. Previous studies have shown the potential beneficial effects of coffee on patients suffering from chronic diseases such as Parkinson’s disease, cardiovascular disease, and cirrhosis. [7–9] Also, Loftfield et al. conducted a large prospective study to demonstrate that coffee consumption may reduce the cause-specific mortality risk from depression, chronic respiratory disease, diabetes, and pneumonia. [10] However, there is not a consensus regarding the effects of coffee on metabolic syndrome. Some studies conducted in Japan have observed that high coffee intake may lower the risk of metabolic syndrome, [4–6] while other studies demonstrated that the consumption of the instant coffee may increase the risk of metabolic syndrome. [11] Furthermore, little is known about the direct association between coffee consumption and insulin resistance.

Several previous studies have identified the association between type II diabetes and coffee consumption. [12–16] For example, upon analyzing the three large cohorts from the United States, Bhupathiraju et al. concluded that coffee consumption over a four-year period may reduce the risk of type II diabetes. [13] However, none of these studies examined the insulinogenic effect of coffee utilizing the Homeostasis Model Assessment of Insulin Resistance (HOMA-IR.)

In this present study, we aim to analyze the correlation between the frequency of coffee intake and the prevalence of metabolic syndrome in a Korean female population. Furthermore, we assess how the association between coffee and metabolic syndrome remains unchanged even after subgrouping the population by variable factors. Lastly, this study evaluates the association between coffee consumption and insulin resistance (HOMA-IR) in Korean female population

## Materials and Methods

### Subject and data collection

The present study used publicly accessible datasets released by Korea Centers for Disease control and Prevention (KCDC), which serves under Korean Ministry of Health and Welfare. KCDC collected the data through Korean National Health and Nutritional Examination Survey (KNHANES), which is a nation-wide survey conducted annually since 1998 to understand the health and nutrition level of the Korean population. [17] KHANES selects the subjects (by household units) using a stratified, multistage probability sampling strategy every year. As KHANES draws the sample group each year (10,000 / 50,000K), there is a very low chance of the same individual being selected more than once. Every participant in the survey provided informed consent. The reviewing process by the Institutional Review Board was not necessary because the usage of the data for the purpose of academic research was already granted to public. Most of the survey questions are in the form of self-administered questionnaire. For biometrics, the qualified examiners at public health centers measured individuals on the day of survey. Kweon at el. has described the details of the data-collecting/physical examination method of KHANES [17]

**Fig 1:**
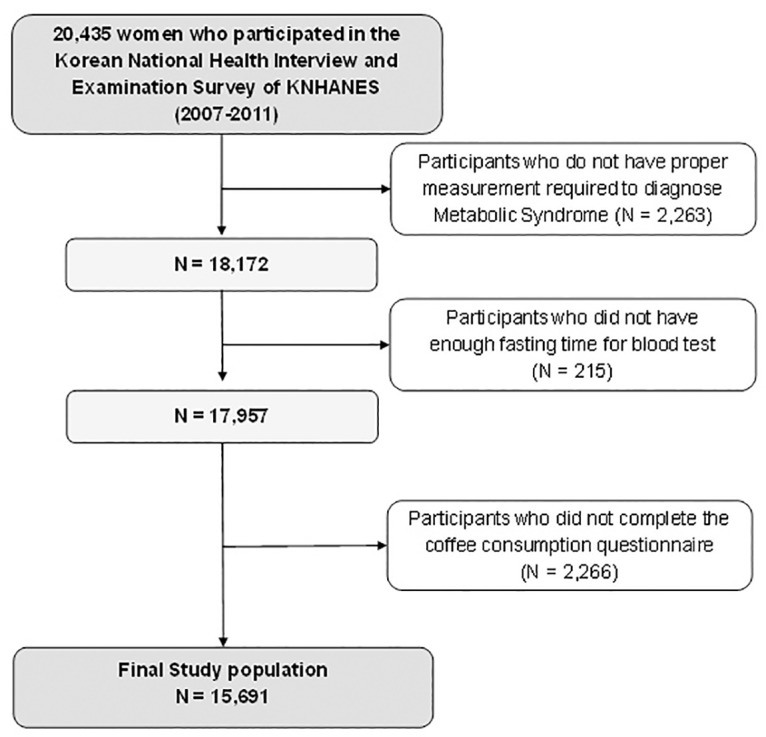
The subjects eligible for study after the exclusion criteria: The Fourth and Fifth Korean National Health and Nutritional Examination Survey. (2007–2011).

Initially, we combined five consecutive years’ worth of data from 2007 to 2011, which summed up to a total of 20,435 women. Then, the exclusion criteria were applied to this sample population to disqualify the individuals who were not eligible for this study. At first, we excluded the subjects (n = 2,263) without proper measurements of the parameters required to diagnose metabolic syndrome. Among the remaining 18,172 subjects, participants without proper fasting time for blood sugar test (n = 215) were also excluded. Additionally, we excluded those who did not provide the information about their coffee-drinking pattern as well (n = 2,266). In summary, 15,691 participants qualified for statistical analysis. (see Fig 1)

In Korean National Health and Nutritional Examination Survey (KNHANES), each survey question usually has more than nine possible response options to choose from. Therefore, when analyzing the result of the survey, we often regrouped together some of these responses, allowing us to perform a more clinically and statistically significant comparison.

### Demographic and life style factors

Every participant in KNHANES completed the questionnaires regarding the demographic factors such as age, sex, and socioeconomic status. For education level, the original survey included nine categories, but we redistributed the subjects into three groups: elementary school graduates, middle or high school graduates, and college graduates or above. Likewise, we regrouped the subjects into four income levels according to their equivalised household income, which was calculated by dividing the total household income by the square root of the number of people in that household.

This study also has collected the information on the various life style factors which may modulate the risk of metabolic syndrome in participants. These possible risk factors for metabolic syndrome are old age, high body mass index (BMI), low physical activity, smoking, and heavy alcohol drinking. [1] The questionnaire provided three response choices for smoking status: current smoker, ex-smoker, and non-smoker. Likewise, we also assigned the subjects into three groups by alcohol consumption level: non-drinker, social drinker, and heavy drinker. This study defined heavy drinkers as those who drank more than 7 drinks in a week or 3 drinks on a single day. According to the National Institute of Alcohol Abuse and Alcoholism (NIAAA), the low-risk drinking habit for developing alcoholic use disorder for women is drinking “less than 3 drinks on any single day and 7 drinks per week” [18]

We assessed the level of physical activity with the International Physical Activity Questionnaire (IPAQ), then categorized the subjects into four groups: those who do not exercise at all, those who exercise with moderate-to-vigorous intensity 1 or 2 times per week, those who exercise with moderate-to-vigorous intensity 3 or 4 times per week, and those who exercise with moderate-to-vigorous intensity more than 5 times per week. [17] The statistical modeling later adjusted for these demographic and lifestyle variables to assess the association between coffee intake level and the risk of metabolic syndrome.

### Parameters of metabolic syndrome and Insulin resistance

This study adopted the clinical identification of metabolic syndrome from American Heart association / National Heart, Lung, and blood Institute’s AHA/NECP-ATPIII guideline. The diagnostic criteria of metabolic syndrome are abdominal obesity, elevated fasting blood sugar level (>100mg/dL), high level of triglyceride (≥150mg/dL), reduced high-density lipoprotein (<40 mg/dL for men, <50mg/dL for women), and high blood pressure level (≥130/85 mmHg). When three or more of the above risks are present in the subjects, they are diagnosed with metabolic syndrome. [19] With regard to receiving any care affecting the blood sugar level and blood pressure level, only those who are getting medical treatments were categorized to have the corresponding risk factor. For abdominal obesity, we used the modified values for Korean populations, developed by Korean Society for the Study of Obesity: waist circumference >90 cm in males or >85 cm in females. [20]

The Homeostasis Model Assessment of Insulin Resistance (HOMA-IR) has been widely discussed as a viable tool to evaluate the level of insulin resistance with a single fasting blood sample. [21] Therefore, we used the HOMA-IR as an insulin resistance index.

### Coffee consumption category

For assessing the frequency of coffee consumption, we utilized the food frequency questionnaires (FFQ) of KHANES. Bae et al. already have found the reasonable reproducibility and validity of FFQ for various nutrients in the Korean population diagnosed with metabolic syndrome. [22] KHANES provide nine response choices reflecting the wide range of coffee consumption, from “almost none” to “3 cups a day.” The other options in-between are as follows: 6–11 cups a year, 1 cup a month, 2–3 cups a month, 1 cup a week, 2–3 cups a week, 4–6 cups a week, 1 cup a day, and 2 cups a day. In order to observe the effect of coffee consumption in a more clinically applicable measure, we reorganized the nine responses into four groups. Reorganization of these responses allowed for the similar distribution of the participants in each group. The first category (n = 3,182) represented the non-consumers and included the subjects that answered “almost none” and “6–11 cups a year.” The second category (n = 3,734) represented those who irregularly consumed coffee, and included from “1 cup a month” to “4–6 cups a week.” The third category (n = 4,061) represented the people who consumed coffee exactly once a day. And the fourth category (n = 4,714) represented the participants who consumed coffee more than once a day. This study did not evaluate the consumption of decaffeinated coffee because such type of coffee is rarely consumed among Koreans.

### Statistical analysis

We carried out statistical analysis utilizing STATA statistical software release 13.0 (STATA Corp., College Station, TX) and SPSS Statistics version 13.0 (IBM Corp., Armonk, NY.) To take the complex stratified multistage probability sampling into account, we evaluated our data using "svy" command in STATA 13.0 for our results to represent the entire Korean female population. Previous studies have demonstrated the validity of using STATA for analyzing a complex “stratified multistage probability sample. [23, 24] We performed a univariate analysis to compare the descriptive characteristics among the groups assigned by the frequency of coffee consumption. We then evaluated the estimated proportions and standard errors of the people with metabolic syndrome in each group. The study accepted all the results with p-value less than 0.05 as statistically significant. Using a negative binomial regression, we evaluated the incidence rate ratios (IRRs) for the prevalence of metabolic syndrome in each coffee consumption quartile, referencing the lowest intake quartile as control. We developed three different models which adjusted for various confounders. Model 1 adjusted for BMI and age only. Model 2 adjusted for socioeconomic factors, lifestyle factors, age, and BMI. The lifestyle and socioeconomic factors taken into consideration included smoking status, drinking habit, exercise level, educational background, and income level. Model 3 then also adjusted for nutrient contents as well as every other variable adjusted in Model 2. Nutrients profile adjusted in Model 3 included total energy intake, percent protein intake, percent carbohydrates intake, percent fat intake, fiber intake, Vitamin C intake, and sodium intake. We also conducted regression analyses several more times after stratifying the sample by age, education level, income level, alcohol history, smoking history, and exercise level. We then ran a sensitivity analysis imputing the missing data with an expectation-maximization (EM) algorithm. We calculated the IRR for metabolic syndrome prevalence using negative binomial regression model, imputing missing information about coffee intake behavior and health examination data. Finally, we compared the least square means of HOMA-IR, adjusted for age and BMI, across the quartiles of coffee intake frequency, using a multiple linear regression analysis to test for a linear trend.

## Results

### Basic statistics of study subject

The total number of study subjects was 15,691. The overall prevalence of metabolic syndrome was 19.7% (n = 3,101). Mean energy intake was 1663.0 kcal/day and mean BMI was 23.0 kg/m^2^. Table 1 demonstrates the basic characteristics of the subjects by the frequency of coffee intake. We observed that the low coffee consumption group exhibited physical inactivity and no alcohol consumption compared to those who drink coffee more often. Both of these behavioral factors have been reported to increase the risk of metabolic syndrome. [2]

**Table 1:** Basic characteristics of study population by frequency of coffee consumption.

	Frequency of coffee consumption				p value*
	~ 0 cup	< 1 cup/day	~ 1 cup/day	> 1 cup/day	
**Age (years)**	40.3 ± 0.7	38.2 ± 0.6	46.2 ± 0.5	44.1 ± 0.3	<0.001
**BMI (kg/m2)**	22.5 ± 0.1	22.7 ± 0.1	23.2 ± 0.1	23.3 ± 0.1	<0.001
**Education (%)**					<0.001
≤Elementary school	45.1 ± 1.5	27.6 ± 1.3	27.5 ± 1.3	18.7 ± 1.0	
Middle/High school	38.9 ± 1.4	50.3 ± 1.5	47.1 ± 1.4	55.8 ± 1.3	
≥College	16.0 ± 1.1	22.1 ± 1.2	25.4 ± 1.2	25.6 ± 1.2	
**Household income**					<0.001
Quartile 1 (low)	21.2 ± 1.2	16.5 ± 1.2	17.5 ± 1.1	12.7 ± 1.0	
Quartile 2	27.4 ± 1.5	23.4 ± 1.4	24.3 ± 1.2	26.0 ± 1.3	
Quartile 3	26.4 ± 1.5	32.1 ± 1.6	29.3 ± 1.3	29.0 ± 1.1	
Quartile 4 (high)	25.0 ± 1.6	28.1 ± 1.6	28.9 ± 1.5	32.4 ± 1.4	
**Smoker (%)**					<0.001
Non-smoker	87.5 ±1.2	88.2 ± 1.1	87.7 ± 0.9	85.3 ± 1.0	
Ex-smoker	6.4 ± 0.8	7.0 ± 0.9	7.5 ± 0.8	5.7 ± 1.0	
Current-smoker	6.0 ± 0.9	4.8 ± 0.7	4.8 ± 0.6	8.9 ± 1.0	
**Alcohol drinking (%)**					<0.001
Non-drinker	39.2 ± 1.4	23.7 ± 1.2	18.3 ± 1.0	12.4 ± 0.9	
Social drinker	45.3 ± 1.4	53.6 ± 1.4	58.4 ± 1.3	59.1 ± 1.3	
Heavy drinker^‡^	15.5 ± 1.2	22.7 ± 1.3	23.3 ± 1.2	28.5 ± 1.2	
**Physical activity (%)**					0.091
None	52.7 ± 1.6	51.1 ± 1.6	51.1 ± 1.4	51.1 ± 1.4	
1~2 /week	19.9 ± 1.2	20.4 ± 1.2	17.8 ± 1.2	16.9 ± 1.0	
3~4/week	12.0 ± 0.9	13.3 ± 0.9	13.0 ± 0.9	14.5 ± 0.9	
≥5 /week	15.4 ± 1.1	15.2 ± 1.0	18.1 ± 1.1	17.5 ± 0.9	
**Daily Diets**					
Total energy (kcal/day)	1636.1 ± 17.1	1664.5 ± 15.0	1620.5 ± 14.1	1714.1 ± 13.0	<0.001
Percent Carbs (%energy)	68.9 ± 0.3	67.8 ± 0.3	69.4 ± 0.3	68.0 ± 0.2	<0.001
Percent Fat (%energy)	17.3 ± 0.2	18.3 ± 0.2	16.7 ± 0.2	17.8 ± 0.2	<0.001
Percent Protein (%energy)	14.1 ± 0.1	14.2 ± 0.1	14.2 ± 0.1	14.1 ± 0.1	0.434
Fiber (g/1000 kcal)	4.0 ± 0.1	4.0 ± 0.1	4.2 ± 0.0	4.1 ± 0.1	0.132
Vitamin C (mg/1000 kcal)	58.2 ± 1.3	62.7 ± 1.4	63.3 ± 1.1	60.3 ± 1.0	0.001
**Psychological variables**					
Sleep (hr/day)	8.2 ± 0.6	8.3 ± 1.0	8.6 ± 0.9	8.0 ± 0.7	0.050

Abbreviations: BMI, body mass index; Carbs, Carbohydrates. All data are weighted to the Korea’s national adult population. Percent Carbs, Fat, and Protein mean the percentage of energy each nutrient contributes to the total energy.

*For categorized variables, (i.e smoking status) the chi-square testing was used to obtained the p-value for each variable. For comparing mean values of some continuous variables (i.e BMI), we instead used ANOVA.

^**‡**^Clinical definition for heavy drinker was those who drink more than 7 drinks in a week or 3 drinks on a single day

In order to evaluate the categorical characteristics among the groups, we conducted a chi-square test. We found that the p-values for chi-square tests on education level, household income, smoking history, alcohol history exhibited less than 0.05, implying the potential relationship between coffee consumption level and these variables. Likewise, in order to analyze the difference of continuous variables (i.e BMI) among groups, we conducted an analysis of variance (ANOVA.) Among these continuous variables, total energy, percent carbohydrates intake, percent fat intake, and the amount of vitamin C consumption yielded p-values lower than 0.05.

### Correlation between the frequency of coffee intake and metabolic syndrome

We found that higher level of the coffee consumption is associated with lower risk of metabolic syndrome in women. Table 2 exhibits that coffee consumption inversely correlates with the prevalence of metabolic syndrome. In Model 1, the IRR between the highest consumption group and the lowest consumption group was 0.67 (95% CI = 0.60–0.74), with the p for trend less than 0.001. Even after adjusting for more potential confounders in model 2 and 3, the overall trend of the inverse relationship did not change. In model 3, which adjusted for life-style factors, socioeconomic status, and nutritional profile, subjects in the highest coffee consumption quartile exhibited 25% reduction in incidence of suffering from metabolic syndrome compared to the control (95% CI = 0.67–0.84; P for trend < 0.001).

**Table 2:** The prevalence of metabolic syndrome by the frequency of coffee consumption.

		Frequency of coffee consumption				p for trend
		~ 0 cup	< 1 cup/day	~ 1 cup/day	> 1 cup/day	
Metabolic Syndrome*						
%, proportion of events (SE)		22.3 (1.3)	17.7 (1.1)	22.7 (1.1)	17.0 (0.9)	
IRR (CI)	Model l^‡^	1.00	0.87 (0.78–0.98)	0.83 (0.75–0.92)	0.67 (0.60–0.74)	<0.001
	Model 2^§^	1.00	0.92 (0.82–1.01)	0.91 (0.82–1.01)	0.74 (0.66–0.83)	<0.001
	Model 3^‖^	1.00	0.93 (0.83–1.05)	0.92 (0.83–1.03)	0.75 (0.67–0.84)	<0.001
**Abdominal obesity**						
%, proportion of events (SE)		22.0 (1.3)	22.3 (1.2)	23.8 (1.2)	24.1 (1.1)	
IRR (CI)	Model 1	1.00	0.97 (0.87–1.07)	0.86 (0.78–0.95)	0.87 (0.80–0.96)	0.001
	Model 2	1.00	0.96 (0.86–1.07)	0.88 (0.79–0.97)	0.89 (0.80–0.98)	0.009
	Model 3	1.00	0.96 (0.87–1.07)	0.87 (0.78–0.98)	0.88 (0.79–0.97)	0.005
**Elevated Fasting glucose**						
%, proportion of events (SE)		18.4 (1.0)	15.8 (1.0)	22.0 (1.1)	19.3 (1.0)	
IRR (CI)	Model 1	1.00	0.99 (0.89–1.11)	0.96 (0.87–1.07)	0.89 (0.80–0.99)	0.019
	Model 2	1.00	1.05 (0.93–1.18)	1.04 (0.93–1.16)	0.97 (0.87–1.08)	0.375
	Model 3	1.00	1.06 (0.94–1.19)	1.06 (0.95–1.18)	1.00 (0.90–1.12)	0.822
**High Triglyceride**						
%, proportion of events (SE)		22.2 (1.3)	18.3 (1.1)	21.9 (1.1)	17.8 (0.9)	
IRR (CI)	Model 1	1.00	0.86 (0.76–0.97)	0.84 (0.76–0.94)	0.73 (0.65–0.82)	<0.001
	Model 2	1.00	0.90 (0.78–1.02)	0.89 (0.79–1.00)	0.77 (0.68–0.87)	<0.001
	Model 3	1.00	0.89 (0.77–1.01)	0.89 (0.78–1.00)	0.77 (0.68–0.88)	<0.001
**Reduced HDL**						
%, proportion of events (SE)		51.4 (1.9)	45.1 (1.8)	49.0 (1.6)	45.4 (1.6)	
IRR (CI)	Model 1	1.00	0.88 (0.81–0.95)	0.92 (0.86–1.00)	0.80 (0.74–0.86)	<0.001
	Model 2	1.00	0.90 (0.83–0.98)	0.98 (0.91–1.06)	0.86 (0.79–0.93)	0.001
	Model 3	1.00	0.92 (0.84–1.00)	1.00 (0.93–1.08)	0.89 (0.82–0.96)	0.019
**High Blood Pressure**						
%, proportion of events (SE)		27.6 (1.3)	20.6 (1.1)	30.7 (1.1)	22.1 (1.0)	
IRR (CI)	Model 1	1.00	0.90 (0.82–0.98)	0.93 (0.86–0.99)	0.79 (0.73–0.86)	<0.001
	Model 2	1.00	0.91 (0.83–0.99)	0.95 (0.89–1.03)	0.83 (0.76–0.90)	<0.001
	Model 3	1.00	0.92 (0.83–1.00)	0.95 (0.88–1.03)	0.83 (0.76–0.91)	<0.001

Abbreviations: IRR, incidence rate ratio; CI, confidence interval; SE, standard error; HDL, high-density lipoprotein. All data are weighted to the Korea’s national adult population.

*Clinical criteria of metabolic syndrome was ≥3 components of the following: 1) abdominal obesity (waist ≥90 cm in men, ≥85 cm in women modified value for Korean); 2) elevated fasting glucose (≥100 mg/dL) 3) high level of triglyceride (≥150 mg/dL); 4) reduced HDL (<40 mg/dL in men, <50 mg/dL in women); and 5) high blood pressure (≥130/85 mmHg);

^**‡**^Model 1: Adjusted for age (10≤ years<20, 20≤ years<30, 30≤ years<40, 40≤ years<50, ≥50 years), BMI (continuous)

^**§**^Model 2: Adjusted for educational background (elementary school or less, middle or high school, college or above), income level (4 quartiles of household income), smoking history (non-smoker, ex-smoker, current smoker), alcohol consumption (non drinker, social drinker, heavy drinker), exercise (none, 1~2/week, 3~4/week, ≥5/week), in addition to the variables adjusted in model 1

^**‖**^Model 3: Nutrient information in addition to variables adjusted in model 2

We have also calculated the IRR for meeting each diagnostic criterion (i.e abdominal obesity) of metabolic syndrome by the frequency of coffee consumption, comparing the strength of each association. Through a negative binomial regression, the study found that coffee consumption inversely correlates with all five of the components defining metabolic syndrome. Also, the p for trend regarding these components, except for high glucose fasting level, were all less than 0.05, suggesting a statistically significant linear trend by the degree of coffee consumption. Among these five diagnostic criteria, the amount of coffee consumption had the strongest negative association with the incidence of high triglycerides. In model 1, the IRR between the highest consumption group and the lowest consumption group was 0.73 (95% CI = 0.65–0.82; p for trend < 0.001). In model 3, the negative trend was not very attenuated upon adjusting for more possible confounders. The prevalence of abdominal obesity among the highest coffee intake quartile was 23% lower than that of the lowest coffee intake quartile (IRR = 0.77; 95% CI = 0.68–0.88; P for trend <0.001).

### Association between the frequency of coffee consumption and metabolic syndrome after subgrouping the population by variable factors

After finding the inverse association between the frequency of coffee intake and the prevalence of metabolic syndrome, we performed several subgroup analyses to overcome the potential pitfall of confounding variables. Bi-partitioning the subjects by possible confounders such as age, education background, income level, smoking status, alcohol consumption status, and physical activity level, we evaluated how these variables may have an effect on the strength of the association between metabolic syndrome and coffee consumption. We again performed a negative binomial regression using Model 3, which adjusted for demographic, behavioral, and nutritional factors. Table 3 below demonstrates the results of these analyses.

**Table 3:** The stratification analysis of association between metabolic syndrome and coffee consumption.

Metabolic syndrome*	Frequency of coffee consumption				p for trend
	~ 0 cup	< 1 cup/day	~ 1 cup/day	> 1 cup/day	
**IRR by modified Model 3**^‡^					
Age < 65 (n = 12,591)	1.00	0.93 (0.77–1.10)	0.97 (0.82–1.14)	0.79 (0.67–0.93)	0.003
Age ≥ 65 (n = 3,100)	1.00	0.95 (0.85–1.05)	0.94 (0.84–1.04)	0.81 (0.71–0.92)	0.004
**IRR by modified Model 3**					
≤Elementary (n = 5,407)	1.00	0.92 (0.83–1.03)	0.92 (0.84–1.03)	0.76 (0.67–0.86)	<0.001
≥Middle/High (n = 10,185)	1.00	0.90 (0.65–1.23)	1.02 (0.78–1.35)	0.79 (0.60–1.04)	0.112
**IRR by modified Model 3**					
Low Income level (n = 7,029)	1.00	0.92 (0.82–1.04)	1.00 (0.88–1.13)	0.81 (0.70–0.92)	0.008
High Income level (n = 8,375)	1.00	0.93 (0.75–1.16)	0.91 (0.75–1.10)	0.75 (0.62–0.91)	0.002
**IRR by modified Model 3**					
Non-smoker (n = 12,739)	1.00	0.91 (0.81–1.03)	0.93 (0.84–1.04)	0.77 (0.69–0.87)	<0.001
Ever-smoker (n = 1,247)	1.00	1.08 (0.71–1.63)	1.08 (0.72–1.62)	0.81 (0.54–1.23)	0.216
**IRR by modified Model 3**					
Non-drinker (n = 3,779)	1.00	1.01 (0.86–1.20)	0.95 (0.82–1.10)	0.77 (0.63–0.93)	0.005
Ever-drinker (n = 11,900)	1.00	0.88 (0.76–1.02)	0.94 (0.82–1.08)	0.77 (0.67–0.89)	0.001
**IRR by modified Model 3**					
No Exercise (n = 8,195)	1.00	0.93 (0.82–1.07)	0.94 (0.83–1.06)	0.75 (0.65–0.87)	<0.001
Exercise (n = 7,402)	1.00	0.93 (0.77–1.12)	0.98 (0.81–1.18)	0.80 (0.66–0.97)	0.036

Abbreviations: IRR, incidence rate ratio; CI, confidence interval. All data are weighted to the Korea’s national adult population.

*Clinical criteria of metabolic syndrome was ≥3 components of the following: 1) abdominal obesity (waist ≥90 cm in men, ≥85 cm in women modified value for Korean); 2) elevated fasting glucose (≥100 mg/dL) 3) high level of triglyceride (≥150 mg/dL); 4) reduced HDL (<40 mg/dL in men, <50 mg/dL in women); and 5) high blood pressure (≥130/85 mmHg);

^**‡**^Modified Model 3: Adjusted for age (10≤ years<35, 35≤ years<60, ≥60 years), BMI (continuous), educational background (elementary school or less, middle or high school, college or more), income level (quartiles of household income), smoking history (non-smoker, ex-smoker, current smoker), alcohol consumption (non drinker, social drinker, heavy drinker), exercise (none, 1~2/week, 3~4/week, ≥5/week), and Nutrient information.

Although the strength of the relationship between coffee intake and metabolic syndrome varied, as exhibited by the different values of IRR in each subgroup analyses, the overall trend of negative correlation did not change in these subgroup analyses with the exclusion of ever-smoker group. Among subjects who smoked or had smoked in the past, we found no definite trend of metabolic syndrome according to the frequency of coffee consumption.

### Adjusted mean of HOMA score according to the frequency of coffee intake

**Fig 2:**
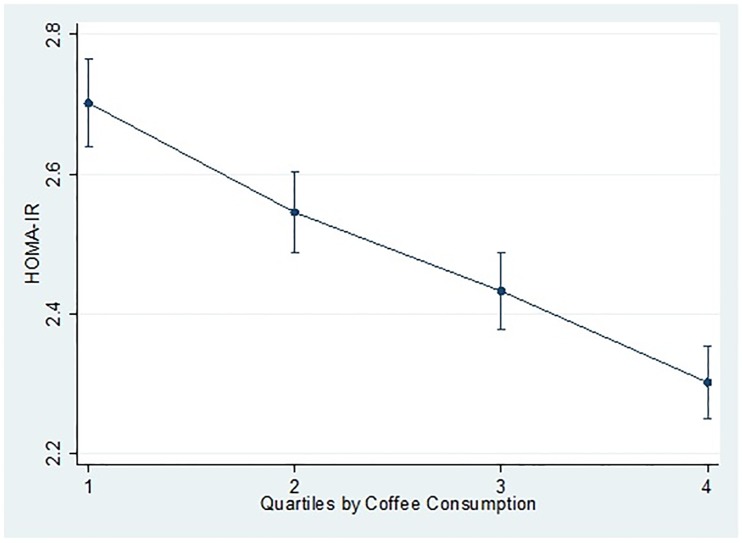
Least-squares means of HOMA-IR according to the frequency of coffee intake. (p for trend <0.001). Abbreviations: HOMA-IR; the Homeostasis Model Assessment of Insulin Resistance. All data are weighted to the Korea's national adult population. Multiple linear regression, model is used, adjusting for age (10≤years<20,20≤years<30,30≤years<40,40≤years<50,≥50 years) and BMI (continuous).

Age- and BMI-adjusted HOMA-IR decreased as the level of coffee consumption increased (P for trend < 0.001). As high HOMA-IR represents a high insulin resistance level, the present analysis suggests the negative correlation between the frequency of coffee consumption and insulin resistance level. Fig 2 below demonstrates the relationship between coffee consumption and HOMA-IR.

### Sensitivity analysis after imputing for missing data

We evaluated that, even after imputing the missing data, the overall trend of negative correlation between metabolic syndrome prevalence and coffee intake frequency did not change. Upon imputation, model 1 found that IRR between the highest consumption group and the lowest consumption group was 0.67 (95% CI = 0.61–0.74), with the p for trend less than 0.001. Table 4 compares the results between our raw data and the imputed data utilizing an expectation-maximization algorithm.

**Table 4:** The prevalence of metabolic syndrome by the frequency of coffee consumption after imputing for missing information.

		Frequency of coffee consumption				p for trend
		~ 0 cup	< 1 cup/day	~ 1 cup/day	> 1 cup/day	
Metabolic Syndrome*						
IRR (CI)	Model l^‡^	1.00	0.87 (0.78–0.98)	0.83 (0.75–0.92)	0.67 (0.60–0.74)	<0.001
	Model 2^§^	1.00	0.92 (0.82–1.01)	0.91 (0.82–1.01)	0.74 (0.66–0.83)	<0.001
	Model 3^‖^	1.00	0.93 (0.83–1.05)	0.92 (0.83–1.03)	0.75 (0.67–0.84)	<0.001
**Metabolic syndrome**						
IRR (CI)	Model 1 (EM imputed)	1.00	0.88 (0.80–0.98)	0.83 (0.75–0.92)	0.67 (0.61–0.74)	<0.001
	Model 2 (EM imputed)	1.00	0.91 (0.82–1.01)	0.90 (0.82–1.00)	0.74 (0.66–0.82)	<0.001
	Model 3 (EM imputed)	1.00	0.94 (0.84–1.04)	0.92 (0.83–1.02)	0.75 (0.68–0.84)	<0.001

Abbreviations: IRR, incidence rate ratio; CI, confidence interval; SE, standard error; EM, Expectation Maximization. All data are weighted to the Korea’s national adult population.

*Clinical criteria of metabolic syndrome was ≥3 components of the following: 1) abdominal obesity (waist ≥90 cm in men, ≥85 cm in women modified value for Korean); 2) elevated fasting glucose (≥100 mg/dL) 3) high level of triglyceride (≥150 mg/dL); 4) reduced HDL (<40 mg/dL in men, <50 mg/dL in women); and 5) high blood pressure (≥130/85 mmHg);

^**‡**^Model 1: Adjusted for age (10≤ years<20, 20≤ years<30, 30≤ years<40, 40≤ years<50, ≥50 years), BMI (continuous)

^**§**^Model 2: Adjusted for educational background (elementary school or less, middle or high school, college or above), income level (4 quartiles of household income), smoking history (non-smoker, ex-smoker, current smoker), alcohol consumption (non drinker, social drinker, heavy drinker), exercise (none, 1~2/week, 3~4/week, ≥5/week), in addition to the variables adjusted in model 1

^**‖**^Model 3: Nutrient information in addition to variables adjusted in model 2

## Discussion

In this study, we found that the participants who reported to drink more than 1 cup of coffee per day had the lowest incidence of having metabolic syndrome compared to non-consumers. We also found a marginally negative correlation between the degree of coffee consumption and Age- and BMI-adjusted HOMA-IR.

As habitual coffee consumption increased in the Asian population over the past few decades, several researchers have tried to identify the effects of coffee on metabolic syndrome, especially in Japan [4–6]: the results of these studies are consistent with our finding. However, these prior studies tend to focus on a specific group of people with particular inclusion criteria, rather than setting the study population to represent the general public. For example, Takami et al., evaluating the 554 adults from Tokushima prefecture, found the coffee consumption inversely correlated with metabolic syndrome. [6] Matsuura et al. showed the similar correlation in the Japanese civil servants. [5] Hino et al. also showed the inverse relationship between coffee and metabolic syndrome in the group of people over 40 years old. [4] Despite the consistent result of inverse relationship between coffee and metabolic syndrome, there was no consensus regarding the association between individual criteria of metabolic syndrome and coffee consumption among these three epidemiological studies. [4–6]

Song et al. showed that coffee intake inversely correlates with the three components defining metabolic syndrome: abdominal obesity, high blood pressure, and elevated fasting glucose level. However, they found no clear relationship between the level of coffee intake and the risk of metabolic syndrome itself. [25] One of the prior studies even found the conflicting result in terms of metabolic syndrome prevalence. Kim et al. found that coffee consumption may increase the risk of metabolic syndrome in the Korean population because the study mainly focused on the consumption of the instant coffee that is usually high in sugar content. [11] On the other hand, our results demonstrated a negative correlation between the degree of coffee intake and metabolic syndrome in women. We present the following hypothesis as the plausible explanation for this discrepancy: the study population of our research is focused on women. To our knowledge, none of these studies in the past have designed their studies to observe how coffee intake affects the risk of metabolic syndrome in Korean women. Women and men have different hormonal systems and metabolic functions. As such, women have higher prevalence of metabolic syndrome than men. [1, 2] Several studies in the past have noted the sex-specific health effect of coffee on women against other diseases [26–30], suggesting the possibility of some biochemical materials in coffee that interacts more specifically with the endocrinal system of women.

The inverse association between coffee drinking and the risk of type II diabetes has been studied by several researchers in the past [12–16]. There are a few candidate components of coffee that are commonly suggested to explain the protective metabolic effect of coffee: caffeine and cafestol. The effects of caffeine in coffee may lower the risk of metabolic syndrome. Caffeine plays a major role in influencing the metabolic functions in our bodies. Previous studies have suggested that consuming caffeine may lower the level of insulin resistance, [31, 32] which coincided with our analysis using HOMA-IR. Also, cafestol, another bioactive molecule present in coffee, may alter the metabolic pathway in human. Through in vitro studies, Mellbye at el. found that cafestol has dual protective effects on type II diabetes. Not only does cafestol stimulate the secretion of insulin, but it also improves insulin sensitivity, increasing the uptake of glucose in muscle cells. [33]

Previous studies have found that old age is one of the strongest yet inevitable risk factors of metabolic syndrome. [34–37] Park et al. have also demonstrated this trend in the Korean population as well. [38] Natural process of aging brings about change in body composition and physiology, resulting in the decline of basal metabolic rate. These changes along with other lifestyle factors make the elderly more prone to metabolic syndrome. Analyzing the subgroup partitioned by age, we demonstrated that even in the sample of old female subjects, who are over 65, the negative correlation between metabolic syndrome and coffee consumption remained consistent. Another strong indicator of metabolic syndrome is high calorie diet. Therefore, for patients with metabolic syndrome, lifestyle modification of weight reduction diet is often recommended. [39] The date from our study suggests that coffee consumption may have a protective effect even in individuals with high calorie diets. Despite higher levels of calorie intake, subjects in the highest coffee intake quartile (≥ 1 cup/day) exhibited the lowest incidence of metabolic syndrome in comparison to the control group. Christinina et al. showed that the highest coffee consumption group also had the highest energy intake level, which was in accordance with our findings. [40]

Due to the cross-sectional nature of the analysis performed on predesigned survey, our study has several limitations. First of all, we were not able to define the causal relationship between metabolic syndrome and coffee consumption. The bimolecular mechanism through which coffee affects the metabolism of women still remains unknown. Also, the study could not evaluate the exact amount of coffee individuals consumed because the questionnaire in KHANES measured coffee by cups only. Despite these limitations, the result of our study is unique for several reasons. First, the present study used well-stratified data to represent the general population of Korea, making it applicable to the majority of healthy Korean women. Secondly, to our knowledge, this study is the first to demonstrate the effects of coffee on insulin sensitivity using the quantifiable measure, HOMA-IR. Lastly, it is noteworthy how the negative correlation between coffee drinking and metabolic syndrome still remained reproducible in various subgroups, encouraging future studies to assess the biochemical and behavioral characteristics of each subgroup more closely.

## Conclusion

In conclusion, the results from this study indicate that the frequency of coffee consumption had an inverse association with the risk of metabolic syndrome and a direct correlation with insulin sensitivity in Korean women. Already very widely and frequently consumed in Korea, coffee may have some protective effects on the metabolic syndrome among Korean women.

Cohort studies with prospective design are warranted to investigate the causality of the relationship due to the certain methodological limitations in our study. Furthermore, future studies need to focus on the mechanism through which coffee affects the metabolism of women.
